# Colorectal Peritoneal Metastases: A Systematic Review of Current and Emerging Trends in Clinical and Translational Research

**DOI:** 10.1155/2019/5180895

**Published:** 2019-04-01

**Authors:** Foteini Stefania Koumpa, Diamantis Xylas, Maciej Konopka, Dieter Galea, Kirill Veselkov, Anthony Antoniou, Akash Mehta, Reza Mirnezami

**Affiliations:** ^1^Department of Surgery, Brighton & Sussex University Hospitals NHS Trust, Eastern Road, Brighton BN2 5BE, UK; ^2^Department of Surgery, Basildon & Thurrock University Hospitals NHS Foundation Trust, Nethermayne, Basildon, Essex SS16 5NL, UK; ^3^Department of General, Endocrine and Gastrointestinal Surgery and Oncology, Poznan University of Medical Sciences, Przybyszewskiego 49, 60-355 Poznan, Poland; ^4^Computational and Systems Medicine, Department of Surgery and Cancer, Faculty of Medicine, Imperial College London, Sir Alexander Fleming Building, South Kensington Campus, London SW7 2AZ, UK; ^5^Department of Colorectal Surgery, St Mark's Hospital, Watford Road, Middlesex, Harrow HA1 3UJ, UK; ^6^Department of Surgery & Cancer, Imperial College London, 10th Floor, QEQM Building, St Mary's Hospital, South Wharf Road, London W2 1NY, UK; ^7^Department of Colorectal Surgery, University College London Hospital (UCLH), 235 Euston Road, Fitzrovia, London NW1 2BU, UK

## Abstract

Colorectal peritoneal metastases (CPM) are associated with abbreviated survival and significantly impaired quality of life. In patients with CPM, radical multimodality treatment consisting of cytoreductive surgery (CRS) combined with hyperthermic intraperitoneal chemotherapy (HIPEC) has demonstrated oncological superiority over systemic chemotherapy alone. In highly selected patients undergoing CRS + HIPEC, overall survival of over 60% has been reported in some series. These are patients in whom the disease burden is limited and where the diagnosis is made at an early stage in the disease course. Early diagnosis and a deeper understanding of the biological mechanisms that regulate CPM are critical to refining patient selection for radical treatment, personalising therapeutic approaches, enhancing prognostication, and ultimately improving long-term survivorship. In the present study, we outline three broad themes which represent critical future research targets in CPM: (1) enhanced radiological strategies for early detection and staging; (2) identification and validation of translational biomarkers for diagnostic, prognostic, and therapeutic deployment; and (3) development of optimized approaches for surgical cytoreduction as well as more precise strategies for intraperitoneal drug selection and delivery. Herein, we provide a contemporary narrative review of the state of the art in these three areas. A systematic review in accordance with PRISMA guidelines was undertaken on all English language studies published between 2007 and 2017. In vitro and animal model studies were deemed eligible for inclusion in the sections pertaining to biomarkers and therapeutic optimisation, as these areas of research currently remain in the early stages of development. Acquired data were then divided into hierarchical thematic categories (imaging modalities, translational biomarkers (diagnostic/prognostic/therapeutic), and delivery techniques) and subcategories. An interactive sunburst figure is provided for intuitive interrogation of the CPM research landscape.

## 1. Introduction

Colorectal peritoneal metastases (CPM) are identified in 5-10% of patients undergoing colorectal cancer resection, and metachronous CPM occur in an estimated 20-50% of patients during follow-up [1–3]. Peritoneal dissemination is associated with abbreviated survival and significantly impaired quality of life. Survival for patients with CPM ranges from 5 months in untreated cases to an estimated 12-18 months with modern systemic chemotherapy (including biologicals) [1–6]. Several recent studies have shown substantially shorter survival with CPM as compared with nonperitoneal colorectal metastases [7]; this is likely owing to biological and histological differences as well as the relative chemoimpenetrability of the peritoneal cavity compared with visceral metastatic sites [7]. The diagnosis of CPM is often made at an advanced stage due to the lack of specific symptoms of peritoneal involvement and the low sensitivity of current imaging techniques. In select cases, the diagnosis of CPM is established at an earlier stage where multimodality treatment in the form of cytoreductive surgery (CRS) and hyperthermic intraperitoneal chemotherapy (HIPEC) can be considered. The combination of CRS and HIPEC was popularized by Sugarbaker et al. in the early 1990s [8], and the oncological superiority of this approach over systemic chemotherapy in isolation has been confirmed by several recent phase II and III clinical trials [9].

Data synthesized from multicentre analyses report median overall survival rates of up to 63% in highly selected patients undergoing CRS and HIPEC [5, 10–13]. Concurrently, the reported 1-year mortality rate and recurrence rate after CRS and HIPEC are estimated to be 13% and 35%, respectively [14]. Several points of discussion emerge from these data; firstly, increased survival is possible in a select group of patients with CPM, and so more accurate modalities are needed with which to identify these patients early on in the disease course; secondly, up to one third of patients offered CRS and HIPEC relapse within 12 months, and therefore, more robust methods for identifying these patients prior to radical treatment should be developed—either with a view to avoiding radical surgery altogether or developing novel ways of modifying the risk of relapse/enhancing the therapeutic response. Beyond these aspects, further improvements in survival for patients with CPM will require a deeper understanding of the molecular processes that drive peritoneal spread and those that dictate peritoneal chemosensitivity. In addition, the techniques of CRS and HIPEC, including approaches to drug selection and drug delivery, have undergone only superficial modification since the original description proposed by Sugarbaker. Extrapolating further from these points, three definable areas emerge as critical future research targets in CPM: (1) enhanced strategies for early detection; (2) identification and validation of translational biomarkers for diagnostic, prognostic, and therapeutic utility in CPM; and (3) development of optimized approaches for surgical treatment as well as more precise strategies for intraperitoneal drug selection and delivery. In the present article, we provide a contemporary narrative review of the state of the art in these three areas.

## 2. Methods

### 2.1. Identification of Studies

An electronic literature search was carried out using MEDLINE (November 2007 to November 2017), EMBASE (November 2007 to November 2017), CINAHL (November 2007 to November 2017), and the Cochrane Library databases. The following medical subject heading (MeSH) terms and keywords were used: “*colorectal*,” “*peritoneal*,” “*metastases*,” “*cytoreductive surgery*,” “*HIPEC*,” and “*intra-peritoneal chemotherapy*.” The “related articles” function was used to broaden the search, and all abstracts, studies, and citations retrieved were scanned for subject relevance. The latest date of this search was November 2017. All potentially relevant publications were retrieved in full text and formally evaluated for study inclusion. Reference lists of all relevant publications were hand-searched for additional studies missed by the search strategy, and this method of cross-referencing was continued until no further relevant publications were identified.

### 2.2. Study Inclusion Criteria and Data Extraction

**Figure 1:**
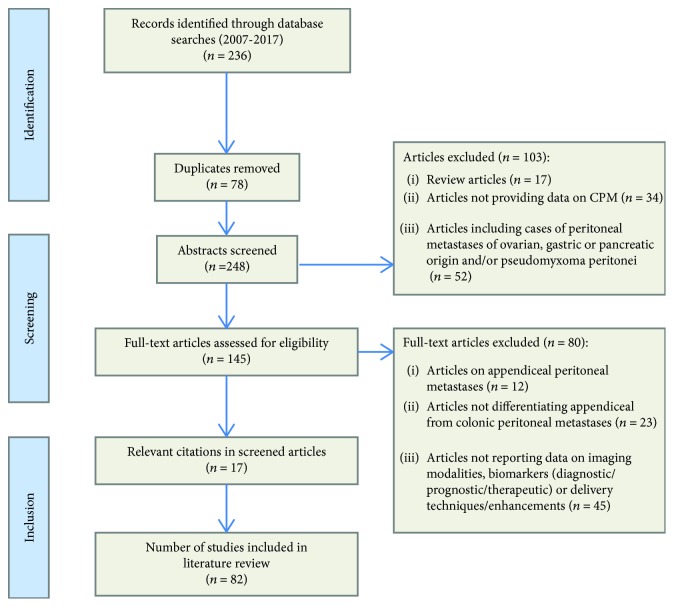
Modified preferred reporting items for systematic reviews and meta-analysis flow diagram outlining study selection strategy.

Study methodology was carried out in accordance with the “Preferred Reporting Items for Systematic Reviews and Meta-Analyses” (PRISMA) recommendations for improving the standard of systematic reviews [15]. Studies that met the following predefined criteria were included in the review process: *Language:* only English language publications were included. *Patient population:* studies had to report outcome data specifically on the use/investigation of one or more of the following: (i) radiological/imaging approaches for diagnosis and staging of CPM; (ii) novel techniques for treatment of CPM including pharmacological and/or surgical approaches—novelty here was defined as any therapeutic approach described and evaluated beyond conventional CRS + HIPEC (defined as complete macroscopic resection of parietal and visceral peritoneal carcinomatosis combined with hyperthermic intraperitoneal chemotherapy); and (iii) biomarkers for CPM diagnosis, prognosis, and therapy. Studies reporting on in vitro or animal model data were considered eligible for inclusion for sections pertaining to novel technical approaches, translational biomarkers, and therapeutic optimisation. For imaging studies, included studies had to provide data on modality-specific sensitivity, specificity, or other metric representative of accuracy of the radiological technique employed. In the event that studies were found to provide data deemed admissible across more than one of these domains (e.g., a study providing data on outcomes for a radiological technique as well as data on therapeutic approach and/or biomarkers), then this data would be included separately under the relevant subheadings in the review process and results. Studies reporting data for CPM as well as peritoneal metastases of other origin(s) were excluded unless specific outcome data pertaining to CPM was disclosed by the authors. *Type of intraperitoneal chemotherapy:* studies reporting on all established and experimental methods of intraperitoneal chemotherapy were included. *Previous treatment:* studies reporting on previously treated patients with CPM were included. Where multiple studies describing the same patient population were identified, the most recent publication was used unless additional information was imparted by earlier work. In cases of doubt, authors were contacted for further information to ensure accuracy or for additional data.  Figure 1 depicts the screening process for selection of potentially relevant studies. Two reviewers (SK and DX) independently extracted the following data from all eligible studies according to a predetermined protocol: first author, year of publication, study location, study type, study time frame, population characteristics, number of subjects, and biomarker investigated (where applicable); radiological/nonradiological technique for detection/surveillance (where applicable); intraperitoneal chemotherapy administration technique used (where applicable); and key findings.

### 2.3. Data Analysis and Data Representation

**Figure 2:**
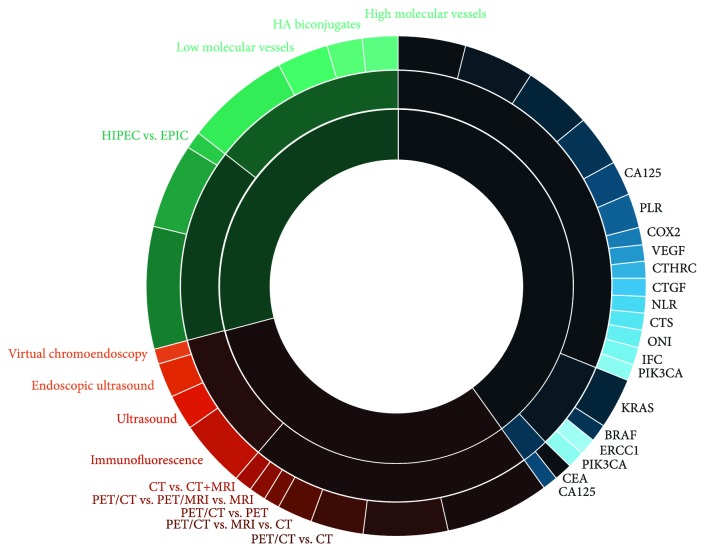
Sunburst figure for visual interpretation of the CPM research landscape. Figure generated using the D3 JavaScript library. A static version of the figure is presented here, and a link to the interactive figure for more targeted interrogation of the available data for given categories and subcategories is provided as supplementary material.

Data acquired via the outlined search strategy was divided into hierarchical thematic categories (imaging modalities, translational biomarkers (diagnostic/prognostic/therapeutic), and delivery techniques) and subcategories. To create a visually intuitive figure for assessment of the CPM research landscape, the number of publications within a given hierarchical category and subcategories has been displayed in the form of a sunburst visualization figure (Figure 2). In this plot, the “upper-level” broad hierarchical categories (imaging/biomarkers/delivery techniques) are represented by the inner figure arches that subsequently divide into more specific subcategories. The circumference of each arch indicates the proportion of publications belonging to a given category/subcategory relative to the rest of the publications in the same hierarchical level. The resulting plot was generated using the D3 JavaScript library.

## 3. Results

### 3.1. Imaging

#### 3.1.1. Radiological Diagnosis and Staging of CPM

Predefined search criteria identified 19 studies evaluating different radiological modalities for detection and staging of CPM (Table 1) [16–34]. Six studies assessed the utility of computed tomography (CT) imaging in this context [19, 23, 31–34] with reported sensitivity and specificity ranging from 11-96% and 49-100%, respectively. The considerable variability in sensitivity with CT has been ascribed to between-study heterogeneity in terms of anatomical site of lesion(s) detected and lesion size [19, 22, 31, 32]. Studies where per-region analysis was performed indicate that areas where CT achieves highest sensitivity are the epigastrium (67-98%) [19, 32], the left upper quadrant (40-86%) [19, 32], and the pelvis (60-73%) [19, 32]. In contrast, regions where CT achieves the lowest sensitivity for lesion detection on CT were found to be the small bowel (8-71%) [19, 23, 32] and adjacent to the ligament of Treitz (20%) [23]. Subanalysis based on lesion size demonstrates a positive correlation between sensitivity of CT for lesion detection and lesion size, with sensitivity ranging from 11 to 70% for lesions under 0.5 cm in size, compared with 90-94% for lesions greater than 3 cm [19, 23, 31]. All three studies [19, 23, 31] used a similar oral and intravenous contrast protocol; however, differences in CT slice thickness, lesion size cut-off values, and lesion site(s) are likely to be responsible for the wide range in sensitivity (11-70% in the case of lesions under 0.5 cm).

In terms of estimation of CPM burden, Koh et al. found that the CT-defined peritoneal carcinomatosis index (CT-PCI) significantly underestimated the disease extent compared to surgical PCI (S-PCI; *p* < 0.001) [19]. However, correlation analysis carried out in two other studies showed a favourable correlation between CT-PCI and S-PCI scores [31, 33]. A recent meta-analysis by Laghi et al. also reported a good correlation between CT-PCI and S-PCI, though the authors did note the potential for CT to underestimate PCI by 12-33% [25]. Several authors have shown that underestimation of PCI with CT is diminished somewhat when a PCI cut-off of 20 is employed [32, 33].

Four [16–18, 22] studies provided data on PET/CT for assessment of CPM. Liberale et al. [22] reported sensitivity and specificity of 85% and 88%, respectively, for detection of CPM using PET/CT. Four studies compared PET/CT to CT [20, 24, 28, 30] with two reporting significantly lower sensitivity with PET/CT compared to CT (82% vs. 91% [24]; 57% vs. 82% [30]). Conversely, a retrospective evaluation of 23 patients with CPM by Bamba et al. [20] demonstrated markedly improved detection accuracy with PET/CT compared to conventional CT (82.6 vs. 30% [20]). In terms of postsurgical surveillance of CPM, Choi et al. showed that PET/CT had higher sensitivity for detecting peritoneal recurrence compared to CT, both compared to histopathological confirmation (100% vs. 85.1%) [28]. In all of these studies, gold standards for confirming diagnosis of CPM and assessing PCI were defined as histological confirmation and surgical PCI estimation, respectively [16–18, 20, 22, 24, 28, 30]. The wide variation in reported sensitivity and moreover the opposing findings reported by some authors regarding the role of PET is difficult to robustly account for. However, at least in part it is likely related to the fundamental fact that PET relies on changes in glucose metabolism, which can vary widely between tumours in a given study cohort; for example, mucinous and signet ring cell carcinomas will tend to exhibit relatively little ^18^F-FDG uptake compared to other histological subtypes.

No studies were identified reporting exclusively on MRI in CPM detection, though four [25–27, 29] were found that compared or combined MRI with alternative diagnostic imaging modalities. Brendle et al. [27] compared the diagnostic accuracy of PET/CT, PET/MRI, and diffusion-weighted MRI (MRI-DWI) and found that conventional MRI alone had inferior diagnostic accuracy compared to when combined with DWI or PET or when compared with CT (diagnostic accuracy of 46%, 47%, 57%, and 66%, respectively). It is interesting to note the very modest increment in diagnostic accuracy with MRI-DWI observed in this study, as there is growing interest in the role of this approach in peritoneal malignancy, which seems difficult to justify based on these findings. Two further studies compared the accuracy of MRI with PET/CT and conventional CT/multidetector CT (MDCT) [25, 26]. Satoh et al. [26] retrospectively evaluated imaging accuracy in patients with peritoneal metastases, of varying primary origins, including colorectal. The authors found that the sensitivity, specificity, and positive predictive values of PET/CT was significantly better than those of MRI or MDCT. However, the meta-analysis performed by Laghi et al. [25], which represents the only pooled analysis of data, failed to show a significant overall difference between CT, MRI, and PET/CT. Nonetheless, the authors did note that when evaluating studies directly comparing CT to PET/CT, sensitivity and specificity for PET/CT were higher than those of CT for detecting peritoneal metastasis (82% vs. 66% and 93% vs. 77%, respectively) [25]. It is likely that a combination of imaging approaches may offer the greatest diagnostic accuracy, and this was recently pointed out by Dohan et al. who found that the addition of MRI to CT for preoperative imaging led to a significant increase in diagnostic sensitivity (CT 54%, CT+MRI 81%; *p* = 0.01) [29].

#### 3.1.2. Intraoperative Imaging and Enhanced Laparoscopy

Staging laparoscopy plays an important role in CPM assessment as it allows direct visualization of the peritoneal surface, tissue biopsy, and PCI estimation. However, Thomassen et al., and a number of other authors, have suggested that open surgery yields significantly higher diagnostic accuracy compared to laparoscopy for CPM [35]. In the present review, we identified 4 studies [22, 36–38] evaluating different techniques with which to enhance minimally invasive peritoneal lesion detection (Table 2). One study reported on the application of virtual chromoendoscopy using the Fuji Flexible spectral Imaging Color Enhancement (FICE) endoscopy system [38], and three studies described the use of fluorescence imaging approaches [36, 37, 39]. The FICE endoscopy system has ten different light wavelength patterns, or channels, which can be customised/reconfigured according to the intended application. In the study by Najah and colleagues [38], the use of the “Channel 2” FICE wavelength showed considerable superiority over standard laparoscopic visualization for detection of peritoneal nodules.

Fluorescence imaging using indocyanine green pigment and intraoperative infrared imaging (ICG-FI and NRIF) was described in 4 studies (Table 2) [36, 37, 39, 40]. Sensitivity for peritoneal lesion detection ranged from 72.4% to 96.9%, with reported specificity ranging from 60 to 100%. When compared with conventional visual inspection of the peritoneal surface, ICG-FI displayed superior diagnostic performance (*p* = 0.027) and resulted in a higher median detected PCI score (10 vs. 7; *p* < 0.001) [37]. However, Liberale et al. [39] importantly noted that this modality was ineffective for detecting mucinous lesions. One small study including 7 human participants [40] trialled a molecular fluorescent agent linked to a tumour target (bevacizumab conjugated to the near-infrared fluorescent dye IRDye 800CW that targets tumour VEGF-A) with reported sensitivity and specificity of 100% and 54%, respectively.

### 3.2. Delivery Techniques

Thirty-two studies were identified evaluating different techniques for intraperitoneal chemotherapy in CPM (Table 3) [41–72]. Sixteen of those studies looked at drug delivery techniques [41, 42, 44–46, 49, 51, 52, 54, 55, 58–60, 62, 67, 68], and 16 looked at novel pharmacological approaches for drug conveyance and therapeutic enhancement [43, 47, 48, 50, 53, 56, 57, 61, 63–66, 69].

#### 3.2.1. Hyperthermic Intraperitoneal Chemotherapy (HIPEC)

Nine studies were identified that evaluated HIPEC related morbidity, mortality, drug penetration, and distribution [46, 51, 52, 55, 59, 60, 62, 67, 68]. Two main HIPEC techniques are employed: the open abdomen (Coliseum) technique and the closed abdomen technique. One study provided data on the open technique [62], 3 reported modifications to the open technique [55, 60, 68], 3 compared the open to the closed approach [46, 52, 59], and 1 reported on outcomes of the closed technique in combination with early postoperative intraperitoneal chemotherapy (EPIC) [51]. Finally, 1 paper described a safe, low-cost delivery method using cardiac bypass pumps as an alternative to the conventional HIPEC instillation circuit [67].

Morbidity and mortality for the open technique ranged from 43.5 to 55% and from 0 to 5.1%, respectively [52, 59, 62]. Spiliotis et al. demonstrated that a positive correlation between mortality and high PCI index, duration of surgery and blood loss, and morbidity was mainly due to pulmonary complications [62]. A number of studies have reported differences in morbidity and mortality with open versus closed delivery approaches [51, 52, 59], but these have not yielded conclusive results. The open technique has the advantage of achieving more even distribution of the chemoperfusate and additionally permits anastomosis of bowel after HIPEC delivery, mitigating the perceived risk this poses to anastomotic integrity. Disadvantages with the open approach include heat dissipation and the risk of personnel exposure to the chemotherapeutic agents. In contrast, the closed technique is associated with uneven drug distribution in the peritoneal cavity but eliminates surgical team cytotoxic drug exposure. The closed approach has also shown superior patient temperature and haemodynamic stability parameters compared with the open approach, perhaps making it a most appropriate choice for frail patients. Interestingly, Facy et al. looked at drug tissue penetration in open and closed techniques [46] and found drug penetration to be significantly higher in the open technique compared to the closed, both when using atmospheric and high intraabdominal pressures for the closed technique.

#### 3.2.2. Pressurised Intraperitoneal Aerosol Chemotherapy (PIPAC)

PIPAC is performed using high CO_2_ pressures (12 mmHg at 37^o^) converting the intended drug, most commonly oxaliplatin, into an aerosol which is sprayed into the peritoneal cavity. Five studies reported data on PIPAC for CPM [41, 44, 49, 54, 58]. These studies present PIPAC results in the palliative setting, in patients with irresectable disease, extensive CPM, and/or those having previously undergone CRS and/or systemic chemotherapy, who were left with residual disease or developed disease recurrence at follow-up [41, 44, 49, 54, 58]. Treatment-associated morbidity ranged from 9.5% to 23%, the mortality from 0% to 6.8% [41, 44, 58]. Median survival post-PIPAC was 15.7 months [44]. PIPAC drug penetration and distribution was found to be 300 *μ*m, mostly concentrated around the micropump, with low penetration in distant areas such as the stomach and subphrenic areas in a swine model [54]. Tumour response to PIPAC was insufficient when used without any additional chemotherapy regimes [58] but ranged from a PCI improvement of 50% to 88% when PIPAC was combined with systemic treatment [41, 44, 49, 58]. The toxicity of PIPAC was assessed by 2 papers and ranged from no renal or hepatic toxicity [58] to low toxicity in 42% of cases with good tumour response [44]. One study assessed the role of PIPAC in CPM for symptom management [41] where symptoms of pain, ascites, and bowel transit disorders resolved following three cycles of PIPAC in 63.3%, 60%, and 45.5%, respectively, with a 3-day median hospital stay.

#### 3.2.3. Early Postoperative Intraperitoneal Chemotherapy (EPIC)

EPIC is administered post-CRS with 5 days of intraperitoneal drug infusion, commonly using mitomycin C and 5-FU. Two studies compared EPIC with HIPEC for CPM [42, 45]. In these studies, EPIC alone was not found to be superior to HIPEC with respect to mortality, morbidity, or cancer recurrence [45]. However, the HIPEC and EPIC combination was shown to achieve greater recurrence-free survival compared with HIPEC alone [42]. The results of the ongoing ICARUS multicentre study comparing outcomes with EPIC and HIPEC in terms of effectiveness and toxicity are eagerly awaited, and this represents the first RCT of its kind in this setting.

### 3.3. Novel Pharmacological Delivery Methods

Our literature review identified 16 studies describing novel pharmacological drug delivery techniques for intraperitoneal chemotherapy (Table 4). These studies can be divided into 3 subcategories: (i) linking drugs to hyaluronic acid (HA) bioconjugates [56, 61], (ii) drug delivery vessels [43, 47, 53, 57, 63], and (iii) hydrogels [48, 50, 64–66, 69]. No comparative study between delivery methods was identified in our literature search.

#### 3.3.1. Linking Drugs to HA Bioconjugates

Two studies were identified describing the use of HA bioconjugates for enhanced IPEC delivery [56, 61]. Hyaluronic acid derivatives were used to increase tumour cell uptake of cytotoxic medication, shown to occur via CD44 receptor binding leading to endocytosis of the drug [56]. Linking the drug to HA led to greater tolerability and lower bone marrow toxicity compared to cytotoxic drug delivery in the free form [56, 61]. Furthermore, an HA-SN38 conjugate drug was shown to be more effective than the free drug by increasing tumour cell drug uptake and having 16 times greater antiproliferative activity in vitro and significantly smaller tumour burden and malignant ascites in vivo [61]. The authors suggest that the lower systemic toxicity of such formulations will allow their use in vulnerable patients and UGTA1 phenotype patients who are at high risk of bone marrow toxicity.

#### 3.3.2. Drug Loading Vessels

Eight papers were identified exploring the use of drug-eluting beads (drug loading vessels that can prolong drug release) [53] and nanoparticles, nanoliposomes, microspheres, or micelles (which increase drug specificity by being internalized by tumour cells) [43, 47, 57, 63, 70–72]. Intraperitoneally placed doxorubicin and mitoxenone drug-eluting beads were shown to continuously release the drug in lower concentrations decreasing side effects and mortality [53] with a similar decline in tumour load compared to free drug. Similarly, drug-loaded microspheres were also shown to decrease tumour volume [47, 57]; doxorubicin microspheres (50-100 *μ*m) were shown to significantly decrease tumour volume compared to blank microspheres and induce coagulative necrosis [57]. Docetaxel microspheres (45 *μ*m) were also shown to significantly decrease CPM tissue Ki-67 markers and significantly increase median survival in animal models [47].

Nanoliposomes are self-assembling particles made of a lipid bilayer that encloses soluble drugs. Due to enhanced permeability and retention in tumour cells, nanoliposomes can achieve selective uptake by tumour cells, increasing antitumour efficacy whilst minimising collateral side effects [73]. In one study, it was found that drug delivery using IV 188Re-liposomes increased survival by 34% (*p* < 0.05) and decreased tumour volume and ascites by 63.4% and 83.3%, respectively, at 7 days after treatment in mice (*p* < 0.05) [70]. In 2007, a phase I study using PLC (doxorubicin encapsulated in liposomes) as part of the HIPEC regime in 29 patients found nanoliposomes to be well tolerated, resulting in a more favourable side effect profile and overall survival of 30.6 months [72].

Expansile nanoparticles (100 nm diameter) were also shown to significantly decrease tumour mass and disease severity score (DSS) compared to free drug and control, with no major systemic toxicities in a CPM mouse model [43]. Biodegradable micelles (25 nm diameter) were also identified as vessels for novel chemotherapeutic agents such as the antibacterial chetomin. Even when using this novel agent, drug micelle delivery significantly decreased tumour volume, tumour vessel length, and branching compared to the free drug in CPM [63]. The chetomin micelles were further used to create a hydrogel that significantly decreased tumour weight compared to micelles and free drug (*p* < 0.01) in a CPM mouse model [63].

#### 3.3.3. Linking Drug to Hydrogels

The potential of cytotoxic drugs imbedded in hydrogels was evaluated in six studies [48, 50, 64–66, 69]. All hydrogels used were mildly cytotoxic at very high concentrations but well tolerated otherwise in animal models. The hydrogels are combined with drug vessels making composite drug delivery systems. Two of the six studies used nanoparticles in hydrogel [48, 64], three used micelles in hydrogel [50, 65, 66], and one used biodegradable microspheres in hydrogel [69]. All hydrogel composites used were thermosensitive, enabling them to remain solid intraperitoneally and offer a slower release drug formulation [66]. They were found to significantly suppress cell growth in vitro [48, 64], increase tumour cell apoptosis [66], and decrease microvessel formation [66]. Four studies also showed that vessel and hydrogel composites significantly decreased tumour nodule weight and number [50, 65, 66, 69] compared with free drug delivery. In animal models, cancer-specific survival was shown to be significantly enhanced in the hydrogel group compared to the free intraperitoneal drug delivery [48, 50, 65, 66]. Furthermore, the combination of microspheres or micelles with a hydrogel was shown to be significantly superior in decreasing tumour weight and number compared to free drug and to the drug in the vessel [66, 69].

### 3.4. Biomarkers

Predefined search criteria identified 24 studies [74–97] evaluating the role of biomarkers in the diagnosis, prognosis, and treatment of CPM (Table 5).

#### 3.4.1. Diagnostic Biomarkers

Five studies [76, 83, 84, 89, 97] were identified assessing the value of CEA, CA125, and Ca19-9 as diagnostic biomarkers in CPM; evaluating their sensitivity and specificity for CPM diagnosis; and comparing them to conventional imaging.

Significantly higher levels of CA125 were detected in patients with CPM, with CA125 concentration positively correlating with CPM tumour volume [89]. Subanalysis based on primary lesion site showed that CA125 concentration differed amongst patients with CPM originating from left- versus right-sided colon primaries (*p* < 0.001). Interestingly, this study reported a similar trend for CEA, but only in female patients. In male patients, CEA levels increased with increasing stage of CRC according to UICC classification, but were not consistently raised in patients with isolated CPM. Huang et al. [89] found CA125 to have higher sensitivity in diagnosing CPM compared with CT imaging. This study again reported inferior diagnostic accuracy and sensitivity with CEA compared to CA125 [89]. However, these findings should be interpreted with caution, as the study by Huang and colleagues is subject to several limitations. Firstly, they employed a CPM staging tool that is not widely used (PD score), and so the generalisability of the results is unclear. Secondly, the study lacks a gold standard on which to base diagnostic accuracy. They have compared CT with tumour marker profiling, and in the case of CPM, neither represent a valid benchmark.

Two studies [83, 97] were identified that evaluated the predictive value of CEA and Ca 19-9 (cut-off used 37.0 U/ml) in diagnosing synchronous peritoneal metastases in patients with CRC. Both demonstrated that elevated levels of Ca19-9 were significantly associated with the presence of CPM, whereas CEA did not retain its significant value in multivariate analysis. Lee et al. also found that intraperitoneal CEA levels were significantly correlated with recurrence and peritoneal metastasis, in patients with negative peritoneal cytology, allowing for a measurement of a marker that could aid in developing stratified follow-up regimens for early detection of CPM in high-risk patients [76].

#### 3.4.2. Prognostic Biomarkers

Seven studies found CEA to be a prognostic marker of overall survival [76, 80, 81, 83, 87, 91, 92]. Cut-off thresholds for CEA showed marked variability between the different studies and ranged from CEA > 5 ng/ml to CEA > 70 mg/l. Irrespective of these differences, all authors found that pre-op CEA levels greater than the assigned cut-off value were associated with poor prognosis, reduced overall survival (OS), and impaired disease-free survival.

Interestingly, Ozawa and colleagues [81] failed to demonstrate the prognostic value of CEA in multivariate analysis but found that preoperative CEA correlates with likelihood of complete cytoreduction (CR0).

The BRAF phenotype in association with CPM was evaluated by 3 studies [88, 90, 96]. Tumours with the BRAF mutation were more likely to present with peritoneal metastases and aggressive biology [98]. Sasaki et al. observed that BRAF V600E mutation was more prevalent in patients with CPM as compared to those without [90].

Bong et al. [91] and Ihemelandu et al. [95] evaluated the platelet-to-lymphocyte ratio (PLR), and this marker was shown to be an independent prognostic factor of poor OS, in levels exceeding 200 and 300, respectively. Patients with a PLR of 150-300 had a median OS of 36 months, and those with a PLR < 150 had an OS of 47 months. PLR was established to be a significant prognostic factor in predicting 5-year OS [72].

Vascular endothelial growth factor (VEGF) was found to be evaluated as a prognostic factor in 3 studies [87, 93, 94]. Chia et al. [87] showed that lower levels of intraperitoneal VEGF at the time of abdominal cavity exploration were associated with improved overall survival in patients with CPM. A similar finding was reported by de Cuba et al. [93] and Sluiter et al. [94] with both studies demonstrating a significant association between high tissue VEGF expression and reduced overall survival in CPM. Sluiter et al. [94] also identified epithelial and stromal VCAN expression as a potential marker of improved overall survival.

The latest study by Ubink et al. [99] showed the relationship between consensus molecular subtype (CMS) and occurrence of CPM. CMS4-positive tumours are more likely to present with peritoneal metastases. Furthermore, those cancers have been associated with a poorer response to anticancer drugs, such as oxaliplatin. However, to confirm its suspected resistance to oxaliplatin, further prospective studies should be conducted.

#### 3.4.3. Therapeutic Biomarkers

Eight studies [74, 75, 78, 79, 86–88, 93] were identified evaluating biomarkers for therapeutic monitoring in CPM. Biomarkers evaluated were ERCC1, TS, VEGF, CTGF, and CRC gene expression.

ERCC1 and TS expression levels have shown some predictive relevance but no clear relationship with regards to response and resistance to 5FU and oxaliplatin containing therapeutic regimens [79]. One study showed *in vitro* chemosensitivity testing to be more effective in predicting clinical response to treatment than the aforementioned biomarkers [79]; however, the expression of these markers demonstrated a direct relationship with clinical response to 5FU and/or oxaliplatin-containing combinations.

Another study [86] demonstrated a significant correlation between high BRCA2 gene expression and BLM gene and protein expression with resistance to mitomycin C (MMC) therapy in peritoneal carcinomatosis.

Five studies [75, 78, 87, 88, 93] have proposed further research into targeted treatments that amplify or reduce biomarker expression or that intervene in pathways which have shown significant association with survival and response to treatment in peritoneal carcinomatosis. Varghese et al. [74] demonstrated distinct gene upregulation of IGF1, HIF1, TIMP2, mTOH, COH17, and MSLN in peritoneal metastasis compared to other metastatic sites, suggesting a role for these molecular targets in peritoneal dissemination of colorectal cancer and furthermore indicating a possible role for these targets in the development of peritoneal surface specific anticancer treatments.

Logan-Collins [75] et al. and de Cuba et al. [93] evaluated VEGF expression levels in patients undergoing CRS and HIPEC and found that high VEGF expression was associated with poor overall survival following treatment, identifying VEGF as a promising treatment target and also a useful marker that could identify patients at risk of early treatment failure. Chia et al. [87] demonstrated that low preoperative intraperitoneal (IP) VEGF levels were associated with improved survival and suggest that bevacizumab, which selectively targets the VEGF receptor, could be selectively used in these patients to improve disease control.

Data in a study by Lin and colleagues [78] demonstrated that CTGF has a role in inhibiting colorectal cancer cell adhesion (a crucial step in peritoneal seeding), highlighting the potential to use CTGF for the development of targeted therapies that dampen cell adhesion and mitigate peritoneal seeding.

## 4. Discussion

Improving survival for patients with colorectal peritoneal metastases (CPM) will require advances in radiological assessment, identification and validation of translational biomarkers, and enhancements to surgical cytoreduction and intraperitoneal drug delivery. The aim of the present study was to provide a systematic overview of recent developments across these three broad research domains; acquired data have been integrated into a sunburst figure, offering a means of interactively evaluating the CPM research landscape. The data provided herein represent a streamlined overview of the available literature on CPM, and this is primarily due to strict methodological adherence to predefined criteria for study inclusion. Studies were only included if they provided quantitative data on performance of radiological techniques, outcome data on novel delivery/treatment methods, or where they investigated disease biomarkers. In addition, studies were only included where data on CPM were specifically provided.

In terms of radiological diagnosis and staging, there remains ongoing uncertainty regarding the optimal imaging modality/combination of modalities and the available literature is highly heterogeneous. Computed tomography is currently the most widely used approach due to its versatility and availability. However, as the studies presented in this review demonstrate, CT appears to consistently underestimate PCI compared with surgical findings and has poor sensitivity for plaque-like nodules and those measuring <0.5 cm [19]. Although MRI, and in particular diffusion-weighted MRI, has gained increasing attention in recent years, there remain practical limitations with this approach due to extended scan duration, patient-related contraindications, and limited capacity of MRI scanning facilities compared with CT. Therefore, at the present time, and despite the aforementioned limitations, CT is generally considered to represent the initial imaging technique of choice for evaluating patients with CPM. One way to offset some of the disadvantages of CT is to introduce formal standardisation of image reporting for peritoneal disease. The introduction of proforma-based reporting has had a significantly positive impact on decision-making in regards to multimodality therapy and operative planning in rectal cancer [104]. Evaluation of CPM extent and distribution would benefit from similar standardisation though this has yet to see widespread implementation. The PAUSE algorithm proposed by Chandramohan and colleagues offers an elegant solution designed to provide a common language for communicating imaging findings in CPM [105]. Briefly, the acronym incorporates: P (primary tumour and peritoneal carcinomatosis index), A (ascites and abdominal wall involvement), U (unfavourable sites of involvement), S (small bowel and mesenteric disease), and E (extraperitoneal metastases). Employing this approach should improve the accuracy of CT reporting in CPM, and widespread implementation will help to improve the consistency of data being accumulated in multinational CPM registries. Besides standardisation of reporting strategy, it is also important to try and firmly establish an optimal protocol for CT imaging of peritoneal disease, and there remains considerable variation in practice across institutions. It is, however, generally accepted that the use of positive or neutral oral contrast medium to opacify the small bowel is useful in evaluating involvement of small bowel and its associated mesentery. Our practice involves multidetector CT image acquisition at section thickness of 3 mm. Patients are given 750-1000 ml of water orally 15 minutes prior to the study, together with intravenous contrast administration. Arterial and venous phase image acquisition is then performed 30 and 60 seconds following contrast injection, respectively. Derived scans are evaluated in protocol-driven fashion in a dedicated complex colorectal cancer multidisciplinary meeting, using axial as well as multiplanar reconstructed images.

Adjunct techniques such as portal venous embolization and associating liver partition and portal vein ligation for staged hepatectomy (ALPPS) have revolutionised treatment options in colorectal cancer liver metastases and similar innovative approaches need to be developed for CPM in the future. With regards to technical advancements, PIPAC represents the most radical proposed departure from standard approaches to intraperitoneal chemotherapy. It is designed to generate an artificial pressure gradient enhancing tissue uptake and permitting homogeneous drug distribution within a closed and expanded abdominal cavity. Early experimental work has demonstrated favourable effects of delivering therapy under pressure of pneumoperitoneum, as this appears to counteract elevated intratumoural interstitial fluid pressures and thus enhances drug uptake. Though PIPAC has until now primarily been reserved for symptom control in the palliative setting, there is some emerging evidence to suggest that it can be utilized in the neoadjuvant setting, with the aim of downstaging disease and facilitating subsequent attempts at radical cytoreduction and HIPEC [49].

The data acquired from this review indicate that strategies for enhancing intraperitoneal cytotoxic effectiveness, via bioconjugation or other approach, remain primarily in the experimental phase of development. Most studies have used in vitro models or in vivo animal models. This branch of research in peritoneal malignancy needs urgently to make the transition into carefully designed prospective, ideally randomized, human studies.

In terms of biomarkers, although a growing number of studies are seeking to evaluate translational biomarkers in CPM, none beyond established tumour markers such as CEA, CA 19-9, and CA 125 have effectively made the transition to clinical use, and current evidence in the literature on the relative diagnostic and prognostic utility of these also seems somewhat contradictory. There is an urgent need to derive and validate biomarkers or biomarker panels capable of (1) defining risk of peritoneal relapse at the time of primary cancer diagnosis, (2) diagnosing disease at the early stages of peritoneal dissemination, and (3) identifying markers capable of predicting likely response to a given intraperitoneal chemotherapeutic agent. This level of translational discovery will call for allied initiatives between peritoneal malignancy institutions and biomolecular phenotyping laboratories, ideally within facilities capable of in vitro modelling as well as in vivo animal models, before initiation of human trials. Emerging disruptive technologies such as the iKnife [106] and the MasSpec Pen [107] offer the potential for real-time intraoperative phenotyping, and this perhaps holds the greatest promise in terms of realising the goal of personalised, “off-the-rack,” selection of intraperitoneal chemotherapy. Simultaneously, developing a better basic understanding of what makes the peritoneum “tick” both in health and in disease should allow the development of more effective imaging biomarkers that selectively localise to the peritoneal surface and reveal disease location and volume more precisely. Moreover, a deeper understanding of the mechanisms that regulate the peritoneum should allow the development of peritoneum-specific targeted therapies that overcome the conventional limitations posed to systemic agents by the plasma-peritoneum barrier. This in turn could improve the likelihood of converting inoperable CPM cases to operable, in the same way that this has been achieved for colorectal cancer liver metastases.

## 5. Conclusion

The present study provides a contemporary “snapshot” of the CPM research landscape and summarises current and emerging trends in practice and also highlights potential areas of unmet need. The combination of more precise radiological detection, optimized therapeutics and a deeper understanding of the biological basis for CPM will lead to improvements in survivorship comparable to those seen over the past few decades in colorectal cancer liver metastases. Tertiary referral units with expertise in peritoneal surface malignancy will likely serve as hubs of translational and clinical research in the future with efforts focused on addressing specific research objectives across the three domains outlined here. Clinical and research pathways for the CPM patients should run in parallel longitudinally to generate meaningful clinical, radiological, and molecular phenotyping data at all steps through the CPM patient journey (diagnosis → staging → treatment → prognostication → surveillance).

## Figures and Tables

**Table 1:** Summary of studies evaluating different radiological imaging modalities for detection and staging of CPM.

Author, year, country	*n*	Study design	Imaging modality (*n*)	Study objective(s)	Sensitivity	Specificity	Other results/comments
Dromain et al., 2008, France [30]	30	PCS	PET/CT (30) vs. CT (30)	To assess and compare the performance of CT and PET/CT in the evaluation of PC	PET/CT 57% CT 82%	The interclass correlation was 0.53 (moderate) between CT and surgery and 0.12 (low) between PET/CT and surgery. Extent of the PC:CT 70% underestimation; PET/CT 80% underestimation	

Koh et al., 2009, Australia [19]	19	PCS	CT	To evaluate the utility of preoperative CT in estimating PCI during the patient selection process	CT demonstrated 11% sensitivity for detection of nodules <0.5 mm. 94% sensitivity in lesions > 5 cm		Miliary/plaque-like peritoneal metastases, as confirmed at time of subsequent surgery were not reliably detect by CT

Franiel et al., 2009, Germany [34]	44	RCS	CT	To investigate whether 1 mm thin slices and MPRs of multidetector CT datasets interpreted in addition to isotropic 5 mm thick slices in one session improve the detection of PC	5 mm 64-91% 1 mm 64-96% MPR 82-100%	5 mm 91-100% 1 mm 91-100% MPR 86-100%	Significant increase in sensitivity (*p* = 0.025) between MPR and 1 mm slices for the least experienced radiologist

Marin et al., 2010, Italy [23]	18	PCS	CT	To prospectively investigate the diagnostic accuracy of a 64-section MDCT for the detection of PM (use of surgery and histopathological findings as the reference standard)	75% (CI 68-84)	92% (CI 85-96)	Sensitivity lesions > 0.5 cm in diameter: 89% (CI 75-97) Sensitivity lesions < 0.5 cm in diameter: 43% (CI 28-56)

Satoh et al., 2010, Japan [26]	237	RCS	PET/CT (107) vs MRI (130) vs MDCT (130)	To compare the diagnostic performances of PET/CT, MRI with and without DWI, and contrast-enhanced MDCT in the detection of peritoneal dissemination	MRI 56% MDCT 76% MRI DWI 84% PET/CT 89%		The positive predictive value of PET/CT (93%) was significantly higher than that of the other three modalities (contrast-enhanced MDCT, 73%; MRI without DWI, 70%; MRI with DWI, 72%)

Esquivel et al., 2010, multicentre [32]	52	PCS	CT	To compare CT defined PCI with surgically defined PCI	Inaccuracies of CT-based assessment of lesion sizes were observed in the RUQ (*p* = 0.004), LLQ (*p* < 0.0005), RLQ (*p* = 0.003), distal jejunum (*p* = 0.004), and distal ileum (*p* < 0.0005) Overall, CT underestimated PCI in 33% of cases compared with surgical assessment		

Duhr et al., 2011, Germany [31]	37	PCS	CT	To compare sensitivity and specificity of CT to surgically defined PCI	50%	62%	Better correlation demonstrated in upper and middle abdominal regions

Berthelot, 2011, France [17]	28	RCS	PET/CT	To assess the performance of FDG-PET/CT examinations for the diagnosis and evaluation of the extent of PC	82%	100%	

Choi et al., 2012, Korea [28]	245	RCS	PET/CT (245) vs. CT (245)	Compare CRC surveillance postsurgery with PET/CT vs. serial CT. Diagnosis confirmed at 6/12 follow-up	PET/CT 100% CT 85.1%	PET/CT 97.3% CT 97%	Overall reported diagnostic accuracy: PET/CT: 97.3% CT 95.8%
Bamba et al., 2012, Japan [20]	23	RCS	PET/CT vs. CT	To compare accuracy of PET/CT and conventional CT in detection of CPM	Overall detection accuracy: PET/CT 82.6% CT 30%		

Pasqual, 2014, Italy [24]	58	RCS	PET/CT (47) vs. CT (58)	To evaluate the accuracy of CT and PET/CT to predict the presence of PM, to quantify the extent by comparing the imaging PCI with intraoperative PCI, and to assess the accuracy of CT and PET-CT of predicting complete CRS	PET/CT 82% CT 91%	PET/CT 67% CT 33%	Correlation between pre-op and intra-op PCI in both CT and PET/CT (*p* < 0.05), but both underestimated the intraoperative PCI and failed to adequately assess all the cases with a PCI value higher than 20 (*p* < 0.05)

Audollent et al., 2015 France [16]	37	RCS	PET/CT	To assess the rate of false-positive findings on FDG-PET/CT in patients with CPM	False-positive findings in 11% of patients—principle causes highlighted as previous surgery resulting in granuloma formation, or previous surgery with foreign body implantation (e.g., synthetic/biological mesh)		

Li et al., 2015, China [21]	1441	Meta-analysis	PET (378) PET/CT (1063)	Pooled analysis to evaluate diagnostic accuracy of FDG PET compared with PET/CT in detecting peritoneal carcinomatosis	PET/CT 84% PET 60%	PET/CT 94% PET 98%	

Flicek et al., 2016, USA [33]	42	RCS	CT	To compare sensitivity and specificity of CT to surgically defined PCI	76%	69%	PPV 85% NPV 56%

Brendle et al., 2016, Germany [27]	74	RCS	MRI ¥ MRI DWI ¥ MRI/PET ¥ MRI/DWI/PET ¥ PET/CT ¥	To investigate the diagnostic performance of different combinations of anatomical and functional imaging techniques in PET/MRI and PET/CT for the evaluation of CPM	Diagnostic accuracy MRI 30% MRI DWI 37% MRI/PET 41% MRI/DWI/PET 55% PET/CT 58%		

Liberale et al., 2017, Belgium [22]	26	RCS	PET/CT	To evaluate the performance of 18fluorodeoxyglucose positron-emission tomography (FDG-PET)/computed tomography (CT) in detection of PC from CRC and correlated the most metabolically active quadrant with the most affected peritoneal area determined during surgery	85%	88%	Correlation of 77.3% of the most scored quadrant in surgery and PET/CT

Kim et al., 2017, Korea [18]	671	Meta-analysis	PET/CT	To evaluate the diagnostic accuracy of FDG PET/CT for detecting peritoneal carcinomatosis	77-93% (95% CI)	89-94% (95% CI)	Across 14 studies (671 patients), the overall sensitivity of FDG PET/CT was 0.87 [95% CI (0.77-0.93)] and a pooled specificity of 0.92 [95% CI; (0.89-0.94)]

Dohan et al., 2017, France [29]	28	RCS	CT (28) CT+MRI (28)	The aim was to determine the incremental value of MRI compared with CT in the preoperative estimation of the peritoneal carcinomatosis index (PCI)	MRI 44% CT 63% CT+MRI 81%		CT+MRI is more accurate at predicting surgical PCI compared with one or other in isolation

Laghi et al., 2017, Italy [25]	934	Meta-analysis	CT MRI PET/CT	Pooled analysis of data to define sensitivity and specificity of CT, MRI, and PET/CT in detection of peritoneal carcinomatosis	CT 83% MRI 86% PET/CT 82%	CT 86% MRI 88% PET/CT 93%	

*n*: number of participants; PCS: prospective cohort study; RCS: retrospective cohort study; MPRS: multiplanar reconstructions; MDCT: multidetector computed tomography; DWI: diffusion weighted imaging; ¥: individual breakdown of scans not disclosed.

**Table 2:** Summary of studies evaluating intraoperative imaging/enhanced laparoscopy approaches in the diagnosis and assessment of CPM.

Author, year, country	*n*	Study design	Modality (*n*)	Study objective(s)	Sensitivity	Specificity	Other study findings
Peter et al., 2009, USA [100]	4	PCS	EUS FNA	To assess the effectiveness of EUS directed FNA for sampling lesions suspicious for peritoneal carcinomatosis	EUS FNA biopsy confirmed diagnosis in all 4 cases and avoided the need for more invasive diagnostic procedures		

Wang et al., 2013, China [101]	153	RCS	USS FNA	To evaluate the diagnostic value and safety of ultrasound-guided percutaneous peritoneal lesion biopsies in patients with ascites and/or abdominal distension with unclear causes	Diagnostic accuracy 92.8% Success rate 100% Sufficient sample acquisition 91.5% Minor complications 11% (No major complications reported)		

Caserta et al., 2013, Brazil [102]	24	PCS	USS FNA	To evaluate the effectiveness of percutaneous USS-guided FNA in establishing a definite diagnosis of peritoneal carcinomatosis	Diagnostic accuracy 79% Sufficient sample acquisition 79% Complications 0%		

Robles-Medranda et al., 2016, Ecuador [103]	5	PCS	EUS FNA	Detection of PC via EUS fine needle-guided peritonoscopy	PC could be visualised and effectively sampled in all patients		

Liberale et al., 2016, Belgium [39]	14	PCS	ICG-FI	(i) To evaluate whether the ICG-FI technique (intraoperative injection) is able to detect peritoneal metastases in patients undergoing CRS (ii) To evaluate the sensitivity of ICG-FI to detect additional subclinical PM not identified during peritoneal inspection under white light	Nonmucinous adenocarcinoma 87.5% Mucinous adenocarcinoma 0%	100%	In 4 of 14 patients (29%), the surgery was modified by intraoperative ICG-FI, which detected additional PM not found using visualization and palpation

Barabino et al., 2016, France [36]	10	PCS	ICG-FI	To evaluate whether the ICG-FI technique (intraoperative injection) is able to detect peritoneal metastases in patients undergoing CRS	72.4%	60%	

Harlaar et al., 2016, The Netherlands [40]	7	PCS	NRIF	(i) Evaluate safety and feasibility of FI-guided surgery using bevacizumab-IRDye 800CW (ii) To define correlation of fluorescence with histopathological findings	100%	54.7	High false-positive rate 47%-despite that PCI was lower by 2.9 points with intraoperative NIRF PPV 53%

Lieto et al., 2017, Italy [37]	4	PCS	ICG-FI	To investigate the role of fluorescence imaging (FI) with ICG (ICG-FI) for detecting CPM	96.9%	PPV: 98.4%. Diagnostic performance of ICG-FI was significantly better than preoperative (*p* = 0.027) and intraoperative conventional approaches (*p* = 0.042) Median PCI score increased from 7 to 10 after ICG-FI (*p* < 0.001)	

Najah et al., 2017, France [38]	561	Feasibility study	FICE	(i) To evaluate the effectiveness of the FICE system in staging laparoscopic assessment of PC (ii) To establish the optimal FICE channel(s) for peritoneal exploration and PC detection	Optimal light wavelengths using FICE for PC detection found to be channels 2, 6, and 9 Channel 2 performed significantly better than white light and other FICE channels in terms of visualization of vascular architecture (*p* < 0.001), differentiation between organs (*p* < 0.001), and detection of PC (*p* < 0.001)		

*n*: number of participants; PCS: prospective cohort study; RCS: retrospective cohort study; EUS FNA: endoscopic ultrasound with fine needle aspirate; USS FNA: ultrasound with fine needle aspirate; ICG-FI: indocyanine green fluorescent imaging; NRIF: near-infrared fluorescence molecular imaging; FICE: Fuji intelligent chromo endoscopy; ¥: individual breakdown of scans not disclosed.

**Table 3:** Summary of studies evaluating intraperitoneal chemotherapy delivery techniques for CPM.

Author, year, country	*n*	Study design	Objective(s)	Morbidity	Mortality	Specific complication(s)	Survival	Other study findings
Elias et al., 2007, France [45]	23	RCS	EPIC vs. HIPEC	EPIC 56% HIPEC 47.8%	EPIC 8% HIPEC 0%	GI fistula rate EPIC 26% HIPEC 0%	5-yr survival- EPIC 28% HIPEC 54%	CPM recurrence rate- EPIC 57% HIPEC 26%

Spiliotis et al., 2009, Greece [62]	39	RCS	Open HIPEC	43.5	5.1	Pulmonary 31% GI fistula 20% Haematological toxicity 16% Postoperative bleeding 11%		Morbidity showed significant positive correlation with PCI (*p* < 0.004), duration of surgery (*p* < 0.001) and intraoperative blood loss (*p* < 0.001)

Chua et al., 2013, Australia [42]	263	PCS	EPIC vs. HIPEC vs. HIPEC + EPIC	Grade III/IV/V HIPEC+EPIC 35.5% HIPEC 43% EPIC 43%	HIPEC+EPIC 1.4%		Median recurrence-free survival: HIPEC+EPIC: 33 mths HIPEC: 19 mths EPIC: 20 mths (*p* = 0.046)	

Halkia et al., 2015, Greece [52]	105	RCS	Open vs. closed HIPEC	Grade III-IV Open 55% Closed 40%	Open 3.3% Closed 0%			Closed technique achieved more stable intraoperative temperature (*p* > 0.05)

Facy et al., France, 2015 [46]	20 ₱	PCS	Open vs. closed HIPEC (evaluating effect of technique on tissue drug penetration and secondarily evaluating effect of technique on clinician exposure to cytotoxic agent(s))	Blood oxaliplatin concentration open: 1.45 vs. closed 0.63 mg/kg; (*p* < .0001) Mean tissue concentration mg/kg: Open 0 cm H_2_O 47.08 Open 25 cm H_2_O: 56.39; (*p* = 0.8398) Closed 40 cm H_2_O 48.57 (*p* = .0053 vs. open, *p* > 0.05 vs. closed 20 mm H2O) No drug identified in inner glove surface				

Lotti et al., 2016, Italy [68]	1	TD	Laparoscopic HIPEC	Proof-of-concept study demonstrating feasibility of laparoscopic HIPEC				
Lotti et al., 2016, Italy [55]	10	CS	Laparoscopy enhanced closed HIPEC (study aimed at evaluating adhesion formation in patients undergoing closed HIPEC)			Adhesion formation in 70% during HIPEC		Adhesions found to develop during closed HIPEC in 70% of patients. The authors suggest that these can hamper even drug distribution

Cravioto-Villanueva et al., 2016, Mexico [67]	10	CS	Modified closed HIPEC	20%	0%	Postoperative ileus 20%	Median survival 30 mths	Median length of stay 15 days

Demtröder et al., 2016, Germany [44]	17	RCS	PIPAC	CTCAE 3 23% CTCAE 1 42%	0%		Mean survival after first PIPAC: 15.7 months	Overall response rate 71%

Girshally et al., 2016, Germany [49]	19	RCS	Evaluating the benefit of “neoadjuvant” PIPAC	Overall survival at 24 mths for patients with CPM 52% Survival in patients with CPM undergoing CRS+HIPEC with and without neoadjuvant PIPAC was 32% and 67%, respectively				

Khosrawipour et al., 2016, Germany [54]	2 **₱**	CS	To assess region-specific drug concentrations in laparoscopic PIPAC	Tissue penetration of drug—300-600 *μ*m with PIPAC. Highest drug concentrations noted around the micropump, penetration (*μ*m): Small intestine: 349 ± 65 RUQ: 349 ± 65 LUQ: 140 ± 26 Distant regions 50 to 150				

Robella et al., 2016, Italy [58]	14	POS	PIPAC	46%	0%			Tumour response assessed via RECIST criteria- 5/14 regression, 2/14 stable

Alyami et al., 2016, France [41]	73	RCS	PIPAC	9.5%	6.8%			LOS—3 days Disappearance of PC-related symptoms: 45-63% PCI improvement: 64.5% of patients Enabling of subsequent CRS+HIPEC 8%

Rodríguez Silva et al., 2017, Spain [59]	30	RCS	Open vs. closed HIPEC	Open: 53% Closed: 15%	Open: 0% Closed: 0%			Closed HIPEC: more stable intraoperative conditions, temperature, and haemodynamic stability (*p* > 0.05)

Gupta et al., 2017, India [51]	33	PCS	CRS+closed HIPEC compared with CRS alone	55.17% Grade III-IV: 10.3%	3.4%	46.1% haematological toxicity	4-year survival: CRS+HIPEC: 58.39% CRS alone: 33.33%	

Sanchez-Garcia et al., 2017, Spain [60]	5₱,	CS	Assessing feasibility and technical aspects associated with laparoscopic HIPEC	Haemodynamic parameters and temperature not significantly different with laparoscopic compared with open CRS and HIPEC Surgical time 240 mins, LOS 6 days				

*n*: number of participants; PCS: prospective cohort study; RCS: retrospective cohort study; CCS: case control study; POS: prospective observational study; OS: overall survival; ₱: porcine study; CS: case series; TD: technique description; CTCAE: Common Terminology Criteria for Adverse Events.

**Table 4:** Summary of studies evaluating novel drug delivery systems for optimized intraperitoneal chemotherapy in CPM.

Author, year, country	*n*	Study subjects	Objective(s)	Delivery technique	Therapeutic response	Survival/viability	Toxicity	Other
Harrison et al., 2007 [72]	29	Human study	To examine the safety and pharmacokinetics of intraperitoneal pegylated liposomal doxorubicin (PLD) used in the context of HIPEC in patients with advanced abdominal-only malignancies	Nanoliposomes		MOS 30.6 months	Grade 3 to 4 complications: 9/29	Increased systemic doxorubicin levels found with use of nanoliposomes up to 24 hrs postperfusion

Lin et al., 2009, China [71]	6	Mouse model	To evaluate the biodistribution and pharmacokinetics of ^111^In-labeled vinorelbine- (VNB-) encapsulated PEGylated liposomes (IVNBPL) after intraperitoneal and intravenous administration	Nanoliposomes	Enhanced drug concentration and penetration into peritoneal surface noted with use of ^111^In-labeled VNB-PEGylated liposomes			

Keese et al., 2009, Germany [53]	16	Mouse model	To compare the therapeutic efficacy of either mitoxantrone and doxorubicin delivered in standard free form with drug delivery using drug eluting beads	Drug-eluting beads	Similar decline in tumour load and tumour volume Increased toxicity with free drug delivery	The authors concluded that bead encapsulation of chemotherapeutic drugs may show the advantage of less toxicity in peritoneal spread of colorectal cancer		

Serafino et al., 2011, Italy [61]	12	Rat model	To provide in vitro and in vivo preclinical data on the antitumour efficacy of ONCOFID™-S, a novel bioconjugate of hyaluronic acid (HA) with SN-38 (the active metabolite of irinotecan)	HA bioconjugate	Significantly decreased ascites and tumour volume with bioconjugation vs. control and vs. free drug (*p* < 0.0001 and *p* < 0.005)		HA-SN38 bioconjugation resulted in equivalent cytotoxicity but 16-fold increase in antiproliferative activity compared to free drug	HA-SN38 bioconjugation demonstrated ability to block cell cycle at G2 in lower concentrations than with free drug Increased tumour cell uptake of HA-linked drug was noted via CD44, which is overexpressed in a wide variety of cancer subtypes including colorectal

Colson et al., 2011, USA [43]	12	Mouse model	To assess the antitumour efficacy of paclitaxel-loaded pH-responsive expansile nanoparticles (Pax-Enp) in vitro and using an in vivo mouse model of peritoneal carcinomatosis	Nanoparticles	Pax-Enp led to a significantly enhanced reduction in tumour volume compared with free drug	Median survival: Pax-Enp: 54 days Free drug: 29 days		

Tsai et al., 2011, China [70]	10	Mouse model	To evaluate the biodistribution, pharmacokinetics, micro-SPECT/CT image, dosimetry, and therapeutic efficacy of ^188^Re-labeled nanoliposomes	Nanoliposomes	Radiotherapeutics with ^188^Re liposomes led to enhanced inhibition of tumour growth and ascites compared with 5-FU *p* < 0.05	Radiotherapeutics with ^188^Re liposomes increased survival by 34.6% compared with 5-FU alone (*p* < 0.05)		

Gong et al., 2012, China [50]	12	Mouse model	To develop a dual drug delivery system of self-assembled micelles in a thermosensitive hydrogel composite to deliver hydrophilic and hydrophobic drugs, simultaneously	Micelles and hydrogels	Dual delivery system comprised of pacitaxel-5FU hydrogel decreased tumour weight more than conventional monotherapeutic drug delivery	Survival: Pacitaxel-5FU 42 days Free drug 35 days (*p* < 0.05)	In vivo experiments in a mouse model showed the dual drug delivery system to be nontoxic and biocompatible	Dual drug delivery system led to enhanced bioavailability (18.7x greater for pacitaxel and 21.6x for 5-FU) compared with conventional delivery

Liu et al., 2013, China [69]	6	Mouse model	To develop a biodegradable and injectable composite drug delivery system using camptothecin- (CPT-) loaded polymeric microspheres (MS) in thermosensitive hydrogel for CPM therapy	Microspheres and hydrogels	Significant reduction in mean number and weight of tumour nodules post therapy with microsphere+hydrogel group: CPT-MS/hydrogel group (19.17 ± 9.64; 0.59 ± 0.20 g) CPT-MS (56.83 ± 14.58, *p* < 0.001; 1.30 ± 0.17 g, *p* < 0.001) Free CPT (93.67 ± 12.96, *p* < 0.001; 2.10 ± 0.17 g, *p* < 0.001) Blank MS/hydrogel (154.67 ± 19.13, *p* < 0.001; 3.33 ± 0.29 g, *p* < 0.001)			

Wu et al., 2014, China [63]	12	Zebrafish and mouse models	To investigate whether counteracting the hydrophobicity of chetomin through encapsulation into polymeric micelles (Che-M) could provide a means of enhancing therapeutic efficacy in CPM compared to chetomin in standard form (Che)	Micelles	Tumour volume, length, vessel branching lower in CheM vs. Che (*p* < 0.01)			% of tumour cells undergoing apoptosis following therapy: Che-M—7.12%, Che—5.75% (*p* < 0.05)

Fan et al., 2014, China [47]	32	Mouse model	Docetaxel-loaded porous microspheres (DOC-MS) vs. free docetaxel (DOC)	Microspheres		Median survival: DOC-MS 33 day; DOC-29 days (*p* < 0.05)		

Montagner et al., 2014, Italy [56]	6	Mouse model	To assess the therapeutic efficacy of two bioconjugates derived from the chemical linking of paclitaxel or SN-38 (the active metabolite of irinotecan), to HA in a mouse model of CPM	HA bioconjugates	(i) In vivo, efficacy of bioconjugates or free drugs against luciferase-transduced tumour cells was assessed by bioluminescence optical imaging and by recording mouse survival. The intraperitoneal administration of bioconjugates in tumour-bearing mice led to improved therapeutic efficacy compared with unconjugated drugs (ii) In vitro, bioconjugates were selectively internalized through mechanisms largely dependent on interaction with the CD44 receptor and caveolin-mediated endocytosis, which led to accumulation of compounds into tumour cell lysosomes			

Fan et al., 2015, China [48]	12	Mouse model	To compare the efficacy of intraperitoneal docetaxel-loaded hydrogel nanoparticles (IPDoc+LL37NPs) compared with free drug in a mouse model of peritoneal carcinomatosis	Nanoparticles and hydrogels	IP Doc+LL37NPs resulted in superior reduction in tumour weight and number of nodules (*p* < 0.05) compared with free drug delivery	IP Doc+LL37NPs led to a significant prolongation of survival in tumour-bearing mice compared with free drug (*p* < 0.001)		

Zhang et al., 2015, China [66]	10	Mouse model	To develop a gel-forming drug delivery system for peritoneal carcinomatosis	Micelles and hydrogels	In vitro: micelle-associated drug delivery demonstrated higher cytotoxicity and apoptotic induction In vivo: hydrogel-associated drug delivery resulted in reduced tumour proliferative activity, increased tumour cell apoptosis, and reduced tumour angiogenesis			

Xu et al., 2016, China [64]	8	Mouse model	To develop a hydrogel nanoparticle—paclitaxel (PTX/PECT) formulation for enhanced IP chemotherapeutic effectiveness in a mouse model of CPM	Nanoparticles Hydrogels	PTX/PECT gel decreased tumour weight vs. control (*p* < 0.01) and vs. free drug (*p* < 0.05)			Half-life of PXT/PECT gel was found to be 17-fold greater than free drug (*p* < 0.001)

Yun et al., 2017, China [65]	72	Mouse model	To develop and test a novel hydrogel drug delivery system through the combination of 5-FU loaded polymeric micelles and cisplatin in biodegradable thermosensitive chitosan hydrogel	Micelles and hydrogels	Use of the chitosan hydrogel as a carrier for cisplatin resulted in a significant reduction in the number and weight of tumour nodules (*p* < 0.05)	Survival CS hydrogel (43 days) Free drug (36 days) (*p* < 0.05)		

Pascale et al., 2017, France [57]	12	Rabbit model	To assess the technical feasibility and oncological efficacy of laparoscopic subperitoneal injection of doxorubicin-loaded microspheres for treatment of CPM in a rabbit model Effect of controlled-release chemotherapy on the growth and viability of CPM rabbits	Microspheres	At 7 days following treatment CPM tumour volume was found to be significantly lower in the doxorubicin-loaded microspheres group compared with control (*p* = 0.0425)	Proportion of viable tumour at 7 days posttreatment Doxorubicin microspheres group 38% Control group 56% (*p* = 0.0202)		

HA: hyaluronic acid; micro-SPECT/CT: micro single-photon emission computed tomography.

**Table 5:** Summary of studies evaluating different diagnostic, prognostic, and therapeutic biomarkers in CPM.

Author, year, country	*n*	Study design	Objective(s)	Biomarker category	Investigated biomarker(s) (including cut-off value where applicable)	Oncological outcome(s)						
						Overall survival	*OS* (2 yr)	Disease-free survival	Hazard Ratio (multivariate analysis of OS)	Sensitivity	Specificity	Other
Varghese et al., 2007, USA [74]	28	RCS	To define variations in gene expression according to site of metastasis in colorectal cancer	Diagnostic	IGF-1 HIF-1	Upregulated in CPM						
					TIMP-2 mTOH COH-17 MSLN	Upregulated in CPM						

Logan-Collins et al., 2008, USA [75]	35	RCS	To assess the prognostic significance of tissue VEGF expression levels in patients undergoing CRS+HIPEC	Prognostic	VEGF	Overall survival following CRS+HIPEC Low VEGF expression vs. high VEGF expression 24.9 vs. 14.65 months (*p* = 0.0179)						

Lee et al., 2009, Korea [76]	234	PCS	To evaluate the diagnostic and prognostic significance of CEA and CA-125 in peritoneal fluid aspirates	Diagnostic Prognostic	CEA ≥4 ng/ml	100% (≤4 ng/ml) vs. 82% (≥4 ng/ml) at 30 months (*p* < 0.001)		88% (≤4 ng/ml) vs. 48% (≥4 ng/ml) at 30 months (*p* < 0.001)	Raised levels of CEA levels in peritoneal fluid were found to be associated with an increased susceptibility to recurrence (*p* = 0.001)			
					CA19-9 ≥37 U/ml	98% (≤37 U/ml) vs. 80% (≥37 U/ml) at 30 months (*p* < 0.001)		76% (≤37 U/ml) vs. 58% (≥37 U/ml) at 30 months (*p* < 0.001)				

Gillern et al., 2009, USA [77]	23	ROS	To investigate patterns of KRAS mutational status in patients with CPM	Prognostic	KRAS wild type vs. mutated	No statistically significant correlations found in survival outcomes for CPM between patients exhibiting wild-type compared with mutated KRAS Prevalence of KRAS mutation in CPM was 48%						

Lin et al., 2010, Taiwan [78]	136	PCS	To assess the prognostic utility of connective tissue growth factor (CTGF) expression levels in primary colorectal cancers at determining peritoneal recurrence susceptibility	Prognostic Therapeutic	CTGF (high expression vs. low expression)	78% (high expression) vs. 25% (low expression) at 60 months (*p* < 0.001)	5-year peritoneal recurrence risks: 6% (high expression) vs. 48% (low expression) (*p* < 0.001) Prediction of IP recurrence OR 0.133 (*p* < 0.003) Mechanism of protective effect of high CTGF expression postulated to be through its ability to inhibit colorectal cancer cell-cell adhesion					

Arienti et al., 2012, Italy [79]	28	PCS	To evaluate potential tissue-based therapeutic biomarkers for determination of likely responsiveness to 5-FU and oxaliplatin based chemotherapy in CPM	Therapeutic	ERCC1	High sensitivity (90%) Poor predictivity of resistance to 5-FU/oxaliplatin-based chemotherapy (56.2%)						
					TS	Low sensitivity (40%) Strong predictivity of resistance to 5-FU/oxaliplatin-based chemotherapy (100%)						

Cashin et al., 2012, Sweden [80]	107	PCS	To investigate a novel means of patient selection for CRS+HIPEC in CPM using serum tumour markers	Prognostic	CEA >70 *μ*g/L	The authors developed a composite predictive scoring system (the Corep score (0-18)) using a combination of the 4 tumour markers, at the cut-off values specified. Retrospective analysis revealed that using employing a Corep cut-off of 6 or more would (1) lead to a reduction in the rate of nontherapeutic laparotomy for CPM from 15% to 7%, (2) increase the rate of radical surgery from 84-88%, and (3) increase median survival from 27.6-34.4 months						
					CA 125 >350 kU/l							
					CA 19-9 >70 kU/l							
					CA 15-3 >50 kU/l							

Ozawa et al., 2013, Japan [81]	921	RCS	To evaluate the correlation between preoperative serum levels of CEA and CA 19-9 and likelihood of R0 resection during CRS for CPM	Prognostic	CEA >5 ng/ml	Preoperative CEA < 5 ng/ml R0 resection rate 35.8% Preoperative CEA > 5 ng/ml R0 resection rate 18.2% (*p* = 0.0038 on multivariate analysis)						
					CA 19-9 >37 U/ml	Preoperative CA 19 − 9 < 37 U/ml R0 resection rate 28.3% Preoperative CA 19 − 9 > 37 U/ml R0 resection rate 18.1% (*p* = 0.1171 on multivariate analysis; N.S)						

Tan et al., 2013, China [82]	106	RCS	To compare CTHRC1 expression in primary colorectal cancer tumour tissue in cases with synchronous peritoneal metastases vs. cases without PM Checked if overexpression of CTHRC1 is associated with poor prognosis	Early diagnostic Prognostic	CTHRC1 expression vs. no expression	Overall survival 31.6 (expression) vs. 57.84 (no expression) *p* < 0.001	CTHRC1 upregulated 7-fold in PM as compared to primary CRC (*p* = 0.0055) Significantly increased incidence of CTHRC1 expression in primary CRC with synchronous CPM compared to cases without peritoneal disease (*p* < 0.001)					

Takakura et al., 2014, Japan [83]	1190	PCS	To evaluate the diagnostic and prognostic value of preoperative CEA and CA 19-9 in CPM	Diagnostic Prognostic	CEA ≥5 ng/ml	Correlation between raised CEA and development of CPM (OR 1.97; *p* = 0.42) Correlation between raised CA 19-9 and development of CPM (OR 27.02; *p* = 0.02)						
					CA 19-9 ≥37 u/ml							

Kwakman et al., 2015, The Netherlands [86]	20	Cell line analysis and RCS	To evaluate genetic markers predicting response to mitomycin-C in patients undergoing CRS+HIPEC	Therapeutic/prognostic	BRCA2	Positive correlation seen between BRCA2 expression levels and resistance to mitomycin C in cell line analysis (*p* = 0.02)						
					BLM	Positive correlation seen between BLM expression levels and resistance to mitomycin C in cell line analysis (*p* = 0.01) Patients with CPM exhibiting high BLM expression also showed significantly inferior survival (32% at 24 months) compared to with those with low BLM expression (80% at 24 months) (*p* = 0.04)						

Chia et al., 2015, France [87]	97	PCS	To investigate the prognostic utility of measuring intraperitoneal VEGF expression levels during CRS for CPM	Prognostic Therapeutic	IP VEGF (T1)	On multivariate analysis, a lower level of intraperitoneal VEGF was associated with improved overall survival In addition a lower intraperitoneal VEGF to intravenous VEGF ratio was associated with improved disease-free survival						

Green et al., 2015, USA [88]	16	RCS	To use IHC profiling to define markers correlating with survival in CPM	Prognostic	COX-2	Increased COX-2 expression demonstrated significant correlation with abbreviated survival in the study cohort (*p* = 0.02)						

Huang et al., 2016, Taiwan [89]	853	ROS	To evaluate the sensitivity and specificity of CEA and CA 125 levels in diagnosing CPM compared with CT	Diagnostic	CEA					75.4%	62.8%	
					CA125					61.4%	89.6%	

Sasaki et al., 2016, Japan [90]	526	RCS	To evaluate the impact of gene expression patterns on survival in patients with CPM versus patients with nonperitoneally disseminated metastatic CRC	Diagnostic Prognostic	BRAF	N.S *p* = 0.73	Incidence of BRAF V600E mutation was significantly higher in CPM patients compared with patients with metastatic CRC without peritoneal surface involvement (27.7% vs. 7.3%; *p* < 0.01). This suggests that BRAF V600E mutational status could be used as a guide to determine presence of occult peritoneal disease or predict likelihood of peritoneal recurrence BRAF mutation demonstrates strong correlation with reduced overall survival in non-CPM metastatic CRC, but not with CPM					
					KRAS	N.S *p* = 0.67						
					PIK3CA	N.S *p* = 0.2						

Bong et al., 2016, Japan [91]	60	PCS	Univariate and multivariate analysis of several prognostic factors with overall survival	Prognostic	PLR < 150 150-300 >300	47 36 5 *p* = 0.034			1.035 *p* < 0.001			In predicting 5-year OS only PLR was significant in multivariate analysis (*p* < 0.001)
					NLR > 4.95	No effect 0.839						
					CEA < 5 >5	37 29 *p* = 0.036			1.022 *p* = 0.125			

Huo et al., 2016, Australia [92]	164	RCS	Prognostic significance of tumour markers on survival in CPM	Prognostic	CEA > 6.5 mg/l				2.46 *p* < 0.01			High CEA or Ca 125 HR 3.34 (*p* = 0.02)
					Ca125 > 16 U/ml				2.23 *p* < 0.01			High CEA and Ca 125 HR 6.57 (*p* = 0.001) Median survival: not reached vs. 22 months

de Cuba et al., 2016, The Netherlands [93]	52	RCS, prospectively maintained database	To evaluate association between IHC markers of angiogenesis and survival in CPM following CRS + HIPEC	Prognostic Therapeutic	VEGF (high vs. low expression)	23.8 vs. 36.1 (*p* = 0.002)			3.8 (*p* = 0.008)	High VEGF expression is associated with abbreviated survival after treatment with CRS and HIPEC		

Sluiter et al., 2016, The Netherlands [94]	65	RCS	To assess the correlation between VEGF and VCAN expression levels and survival in CPM	Prognostic	VEGF High vs. low	29.3 vs. 38.3 (*p* = 0.035)			0.315 (*p* = 0.012)	VEFG and VCAN expression, independent prognostic factors for OS High VCAN expression associated with improved cytoreductive outcome (greater likelihood of compete cytoreduction/R0; *p* = 0.003)		
					VCAN High vs. Low	36.8 vs. 20.1 (*p* = 0.19)			2.584 (*p* = 0.042)			

Ihemelandu et al., 2017 USA [95]	123	RCS	To investigate the prognostic value of inflammation based prognostic scores	Prognostic	Platelet-lymphocyte ratio (PLR) >200	Worse OS (*p* = 0.020)						
					CA 19-9 >500	Worse OS (*p* < 0.0001)

Massalou et al., 2017, France [96]	84	RCS	To perform IHC profiling on tissue samples acquired during CRS+HIPEC and to determine the prognostic utility of these in CPM	Prognostic	KRAS Wild-type vs. mutated	35.7mths vs. 51.3 mths (*p* = 0.22)		11.6mths vs. 17.5 mths (*p* = 0.15)				
					BRAF Wild-type vs. mutated	42.2mths vs. 32.2 mths (*p* = 0.78)		13.6mths vs. 11.6 mths (*p* = 0.54)
					MSI Absent vs. present	35.7 vs. 85 (*p* = 0.13)		12.4 vs. 24.9 (*p* = 0.01)

Kaneko et al., 2017, Japan [97]	395	RCS	To determine the diagnostic and prognostic utility of preoperative serum CEA and CA 19-9 levels patients with CRC for prediction of synchronous PM	Diagnostic	CEA > 5 ng/ml	Associated with synchronous PM *p* = 0.038 (univariate); N.S on multivariate analysis						
					CA 19-9 >37 U/ml	Associated with synchronous PM *p* < 0.001 (univariate), and OR 5.03, *p* = 0.02 (multivariate)						

*n*: number of participants; LRM: literature review and meta-analysis; PCS: prospective cohort study; RCS: retrospective cohort study; ROS retrospective observational study; i.p.: intraperitoneal; PM: peritoneal metastasis; IHC: immunohistochemical.

## Supplementary Materials

Supplementary Materials Interactive figure for visual interpretation of the CPM research landscape. The available data for the three main categories and their respective subcategories is represented in the form of a sunburst diagram.
