# Supplementary material for: Colorectal Peritoneal Metastases: A Systematic Review of Current and Emerging Trends in Clinical and Translational Research
Source: Gastroenterol Res Pract. 2019 Apr 1;2019:5180895. doi: 10.1155/2019/5180895 (PMC6466888; doi:10.1155/2019/5180895)
Supplement: Supplementary Materials — Interactive figure for visual interpretation of the CPM research landscape. The available data for the three main categories and their respective subcategories is represented in the form of a sunburst diagram. [file 5180895.f1.zip › index.html]

Publications


of published articles

Legend
